# Echocardiography in the diagnosis left ventricular noncompaction

**DOI:** 10.1186/1476-7120-6-64

**Published:** 2008-12-23

**Authors:** Ze-Zhou Song

**Affiliations:** 1 Department of Ultrasound, The First Affiliated Hospital, College of Medicine, Zhejiang University, Hangzhou, PR China

## Abstract

Echocardiography is the method of choice to establish a diagnosis and determine a treatment plan for patients with noncompaction of ventricular myocardium (NVM). The 2-dimentional echocardiography, 3-dimentional echocardiography, color Doppler echocardiography and contrast-enhanced echocardiography are of critical importance for diagnosis and family screening of NVM.

## Introduction

In normal human hearts of children and adults the left ventricle (LV) has up to 3 prominent trabeculations and is, thus, less trabeculated than the right ventricle [1,2]. Rarely, more than 3 prominent trabeculations that is the so-called LV noncompaction of ventricular myocardium (NVM) can be found at autopsy and by various imaging techniques including echocardiography and MRI etc. in the LV.

**Figure 1:**
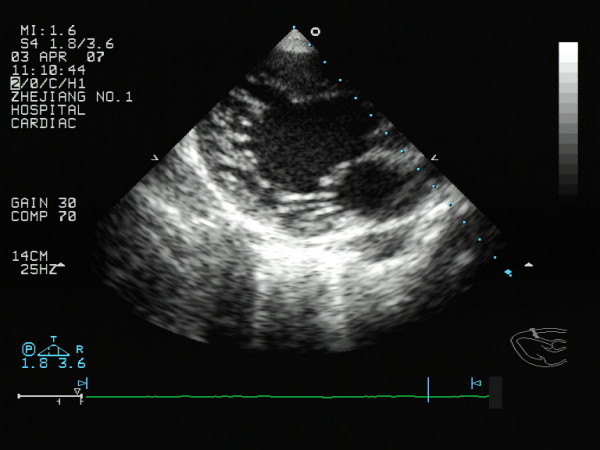
**left ventricular long axis view showing the trabeculationsof the left ventricular wall and deep intertrabecular recesses (arrow) by two-dimension echocardiograms**.

NVM is recently included in the 2006 classsification of cardiomyopathies as a Genetic Cardiomyopathy [3]. NVM occurs because of a disorder of endomyocardial morphogenesis that results in a failure of trabecular compaction of the developing myocardium [4]. In adult patients one or more segments, especially the apical, mid-lateral and mid-inferior regions, of the left ventricle, and sometimes both ventricles, are characterized by numerous sinusoids or trabeculae that are excessive in number and abnormal in prominence and by deep intratrabecular recesses covered by endothelium that exhibits continuity with ventricular endocardium(Figure 1). Numerous modalities have been used in the description, characterization, and diagnosis of NVM including, but not limited to, magnetic resonance imaging, two-dimensional echocardiography (2DE), contrast-enhanced 2DE, and angiography [5,6]. 2DE is by far the most commonly used diagnostic modality. On the basis of echocardiographic studies, the prevalence of NVM has been estimated at 0.05% in the general population [7]. Therefore, the following review aims to give an overview about the current knowledge and controversial issues regarding echocardiography in the diagnosis and management of NVM.

## Diagnosis

### Diagnostic criteria

There are 2 sets of echocardiographic criteria for IVNC diagnosis: the Jenni criteria, which stress the presence of a 2-layered structure, and the Chin criteria, which focus on the depth of the recess compared with the height of the trabecula [8,9]. In both sets, it is important that there are no other cardiac structural abnormalities, such as semilunar valve obstruction or coronary artery anomalies. A comparison of these 2 sets of criteria is presented in Table 1[10].

**Table 1:** Echocardiographic Criteria for the Diagnosis of NVM

Chin Criteria (1990)	Jennni Criteria (1999)
Absence of any other coexisting cardiac structural abnormality	Absence of any other coexisting cardiac structural abnormality
Numerous, excessively prominent trabeculations and deep intertrabecular recesses	Numerous, excessively prominent trabeculations and deep intertrabecular recesses
Views: parasternal long axis, subxyphoid, and apical	Views: parasternal short axis, and apical
Focus on depth of recesses	Focus on a 2-layer structure
Measured in end-diastole	Measured in end-systole
Ratio of distance from the epicardial surface to the trough of the trabecular recesses and distance from the epicardial surface to peak of trabeculation ≤ 0.5	Ratio of thick noncompacted layer to thin compacted ≥ 2 Perfused intertrabecular recesses suplied by intraventricular blood on color Doppler analysis

### 2DE Diagnosis of NVM

2DE is by far the most commonly used diagnostic modality. However, recent studies have found that the diagnosis of this disorder is often delayed because of difficulty in elucidating the diagnostic findings [11]. Frequently, multiple 2DE studies are required before the diagnosis is made. 2DE provide excellent visualization of left ventricular cavity, but are not necessarily adequate for thorough inspection of myocardium and endocardium of entire left ventricular. The prominent trabeculations that characterize NVM may curve into left ventricular cavity in a manner that challenges the planar cuts that are inherent to 2DE. These limitations of 2DE may lead to misinterpretation of prominent trabeculations as "false tendons," and may also lead to underestimation of the severity of NVM. As a result, the 2DE diagnosis of NVM is often not made until patients had several 2DE. Ichida et al. [12] found that the diagnosis was missed altogether in well over half of their study population of children, and similarly, Baker et al. [13] report that three cases underwent an initial 2DE that did not confirm NVM which may be due to the nonuniform nature of NVM.

### Color Doppler echocardiography Diagnosis of NVM

Occasionally, the affected myocardial segments are hypokinetic, contrary to certain forms of apical cardiomyopathy that can otherwise mimic NVM. However, differential echocardiographic diagnosis includes prominent normal myocardial trabeculations, false tendons and aberrant bands, cardiac tumors, and left ventricular apical thrombus. In these cases, the evidence of the direct blood flow from the ventricular cavity into deep intertrabecular recesses via color Doppler echocardiography analysis is helpful for the differentiation of NVM from other echocardiographic abnormalities in which this finding is not observed [8].

### Three-dimensional echocardiography (3DE) Diagnosis of NVM

3DE allowed for accurate diagnosis, detailed characterization, and description of the extent of the affected myocardium in NVM in all four patients of this case series by Baker et al. [13]. Entire trabecular projections and intertrabecular recesses were visualized simultaneously and the distinction between the compact and noncompact LV myocardium was easily demarcated. Wall motion abnormalities were demonstrated simultaneously for large portions of myocardium. In contrast to 2DE, 3DE provides for pyramid-shaped datasets that encompass the entire left ventricular. Specifically, left ventricular can be sectioned in userselected planes and an unlimited number and angles of such planes can be used. Intracavitary echodensities that are suspicious for trabeculations can be tracked in multiple directions from base to apex. The resulting images provide the ability to visualize an entire trabeculation. The deep intertrabecular recesses and the distinction between the prominent noncompact left ventricular myocardium and the thin compact layer of left ventricular myocardium can also be well demonstrated [13].

### Contrast echocardiography Diagnosis of NVM

Conventional echocardiography is the diagnostic modality of choice for INVM. In classic cases, the diagnosis of NVM is not difficult. However, when noncompaction is subtle or incomplete, disorders such as prominent normal myocardial trabeculations, hypertrophic cardiomyopathy, dilated cardiomyopathy, and left ventricularapical thrombus should be included in the differential diagnosis. Conventional echocardiography has some diagnostic limitations in obese patients or patients with lung disease who have poor acoustic windows.

Currently, echocardiography with various contrast agents is being used to enhance endocardial border delineation [14,15]. Koo et al. [16] performed transpulmonary contrast echocardiography with intravenous injection of PESDA (a sonicated mixture of perflourocarbon [8 cc], 5% albumin [4 cc], and 5% dextrose water [8 cc]). Opacification of ventricular cavity with a contrast agent clearly defined the endocardial border, and the authors obtained better images with this method than with conventional transthoracic 2DE. Therefore, when conventional echocardiographic images are poor or diagnosis is uncertain, contrast echocardiography can be helpful. This procedure can be performed without further patient discomfort, and can save time and money. Apical swirling or nonuniform distribution of contrast may simulate endocardial trabeculae, especially in patients with low ejection fraction. Higher doses of contrast or the use of new imaging techniques, such as power Doppler, can be helpful in these situations [17,18]. Therefore, the above-mentioned study should be performed in the future.

### Echocardiography in family screening of NVM

The familial character of LVNC has been recognized and probably up to 44% of patients do have affected family members. Although there are no systematic investigations on the familial occurrence of LV NVM, there are single reports that demonstrate LV NVM to occur in multiple family members. Family screening may unmask affected family members for primary prevention including anti-coagulation and implantable cardiovertor defibrillators (ICD) therapy, especially to early diagnosis of asymptomatic patients of NVM. 2DE, 3DE, Contrast echocardiography could all be the pretty good diagnostic modality of choice for NVM, which need to be further studied.

### Differential diagnosis of NVM

The apical and midventricular segments of both the inferior and lateral wall are affected in more than 80% of patients [8,19]. Involvement of the midventricular anterior wall, basal segments, and septum is much less frequent [8]. The segmental appearance is an important feature, because prominent trabeculae may also be seen in hypertrophied hearts [10].

Although echocardiography has been the diagnostic test of choice for noncompaction, other modalities have been used for the diagnosis, including contrast ventriculography [20], computed tomography [20], and MRI [6]. MRI provides good correlation withecho for localization and extent of noncompaction and is useful in cases with poor echocardiographic image quality [21]. In addition, the demonstration of differences in MRI signal intensity in noncompacted myocardium may help identify substrate for potentially lethal arrhythmias [22]. Although the diagnostic value of cardiac MRI seems to increase in parallel with technical refinements, echocardiography is generally considered the gold standard for the diagnosis of NVM. In most cases, cardiac MRI offered no additional information compared with echocardiography except for the detection of a thrombus which was hidden in the spongelike myocardium of NVM [23]. Cardiac MRI guarantees more operator independency and is less affected by technical limitations than echocardiography, but there has been no large-scale study comparing the 2 modalities [23].

Occasionally, the affected myocardial segments are hypokinetic, contrary to certain forms of apical cardiomyopathy that can otherwise mimic IVNC. However, differential echocardiographic diagnosis includes prominent normal myocardial trabeculations, false tendons and aberrant bands, cardiac tumors, and left ventricular apical thrombus. In these cases, the evidence of the direct blood flow from the ventricular cavity into deep intertrabecular recesses via color Doppler analysis is helpful for the differentiation of IVNC from other echocardiographic abnormalities in which this finding is not observed [8,23].

## Conclusion

In conclusion, echocardiography is the method of choice to establish a diagnosis and determine a treatment plan for patients with NVM. Despite the widespread use of echocardiography, NVM is commonly misdiagnosed because of the lack of knowledge of this disorder, with a significant negative impact on the prognosis of these patients. Therefore, it is still important to make echocardiographers more familiar with this condition and its pathology.

## Competing interests

The authors declare that they have no competing interests.
